# EPDR1 correlates with immune cell infiltration in hepatocellular carcinoma and can be used as a prognostic biomarker

**DOI:** 10.1111/jcmm.15852

**Published:** 2020-09-15

**Authors:** Ruochan Chen, Yiya Zhang

**Affiliations:** ^1^ Hunan Key Laboratory of Viral Hepatitis Xiangya Hospital Central South University Changsha China; ^2^ Department of Infectious Disease Xiangya Hospital Central South University Changsha Hunan China; ^3^ Department of Dermatology Xiangya Hospital Central South University Changsha China; ^4^ National Clinical Research Center for Geriatric Disorders Xiangya Hospital Central South University Changsha China

**Keywords:** biomarker, EPDR1, hepatocellular carcinoma, immune cell infiltration, prognostic

## Abstract

Hepatocellular carcinoma (HCC) has high mortality rate and is a serious disease burden globally. EPDR1 (ependymin related 1) is a member of piscine brain glycoproteins and is involved in cell adhesion. The gene expression, prognostic, and clinicopathological related data for EPDR1 were obtained from multiple transcriptome databases. Protein level of EPDR1 in HCC was verified using human protein atlas and CPTAC databases. EPDR1 co‐expressed genes were identified using LinkedOmics. Functional analysis of the co‐expressed genes was performed using gene set enrichment analysis, Gene Ontology, and KEGG. Statistical analysis was conducted in R. The relationship between EPDR1 expression and immune cell infiltration was analyzed using TIMER and CIBERSORT. The expression of EPDR1 was found to be significantly higher in HCC than in normal tissues. Further, EPDR1 level was correlated with advanced stage of HCC. EPDR1 was associated with multiple signaling, as well as cancer and apoptotic pathways. Further, EPDR1 expression was significantly correlated with purity and infiltration levels of various immune cells as well as immune signatures. This is the first study to report the role of EPDR1 in HCC. EPDR1 can be used as a novel prognostic biomarker as well as an effective target for diagnosis and treatment in HCC.

## 1 INTRODUCTION

Primary liver cancer (PLC) is one of the malignant tumors of the digestive system, which poses a huge threat towards human health; hepatocellular carcinoma (HCC) is the major histological type of PLC.^1^ Globally, HCC ranks sixth in the number of incidences and second in the mortality rate among all malignancies.^2^, ^3^ Moreover, the number of HCC incidences is rising, and it is expected that the annual incidences will be beyond 1 million by 2025.^4^, ^5^ Currently, the diagnosis of HCC is mainly based on the detection of serum alpha‐fetoprotein (AFP), B‐ultrasound, and computed tomography imaging. However, the misdiagnosis and missed diagnosis rates are very high.^6^, ^7^, ^8^ Owing to the insidious onset, poor tumor biological behavior, and rapid progress of HCC, most patients reach advanced stage of the disease and miss the opportunity for radical treatment. Despite the advances in research related to HCC biomarkers, in recent years, lack of specific markers for tumor sub‐types, disease stages, or prognosis remains an important gap in the understanding and treatment of HCC. Further, the prognosis of HCC patients is very poor, and the 5‐year survival rate is as low as 30%.^9^, ^10^ Therefore, identifying specific and sensitive biomarkers related to early diagnosis, treatment, and prognosis of HCC is of great significance.

The ependymin‐related 1 (EPDR1) gene encodes for a protein that is similar to ependymins, which belong to a family of piscine brain glycoproteins.^11^ Ependymins, encoded by *Epd* genes, are transmembrane proteins that play a crucial role in adhesion of neural cells.^12^ For the first time, mammalian ependymin‐related transcript was discovered in 2001 in two colorectal cancer (CRC) cell lines,^13^ and was designated as UCC1 (upregulated in colon cancer 1). Following this discovery, other studies identified and characterized the expression of MERP1 (mammalian ependymin‐related protein 1) in hematopoietic cells, non‐hematopoietic tissues, and in several malignant cell lines and tissues,^14^, ^15^ which was later confirmed to be UCC1, and currently known as EPDR1 gene. EPDR1 is highly expressed in the brain and also detected in other tissues, such as muscle, heart and in extracellular fluids.^14^, ^15^, ^16^, ^17^, ^18^

The function of EPDR1 is not well known, although several studies have reported its differential expression and single‐nucleotide polymorphisms associated with its locus in different pathological and developmental processes, which are known to affect the cell adhesion.^19^, ^20^, ^21^, ^22^, ^23^, ^24^ Furthermore, genetic variants in EPDR1 have been reported to be linked to several diseases, including Dupuytren's disease^21^, ^25^, ^26^ and primary angle closure glaucoma^23^, ^27^; however these observations do not provide obvious insight into the molecular functions of the protein. In 2016, Valiente et al reported that EPDR1 and its spliced isoforms are differentially expressed in human CRC cell lines, and the up‐regulation of EPDR1 in human colorectal cancer was reported to promote cell growth, proliferation, and invasiveness.^28^ Genome‐wide DNA methylation profiling studies revealed that methylation‐mediated epigenetic silencing of EPDR1 may play an important role in preventing CRC progression.^29^ In this context, exploring the role of EPDR1 in the onset and/or development of tumors is particularly appealing.

Our study reported that EPDR1 correlates with immune cell infiltration and can be used as a prognostic biomarker in hepatocellular carcinoma. Gene Ontology (GO) and Kyoto Encyclopedia of Genes and Genomes (KEGG) pathway analyses of EPDR1‐coexpressed genes were performed to explore the associated processes and pathways. In addition, relationship between EPDR1 expression and tumor‐infiltrating immune cells, as well as immune‐related genes was explored through multi‐dimensional analysis. The current study will provide novel insights into the underlying mechanisms associated with HCC development, thus help in discovering novel potential targets and strategies for HCC diagnosis and treatment.

## 2 MATERIALS AND METHODS

### 2.1 EPDR1 expression in HCC from different databases

The expression of EPDR1 in HCC was investigated using the datasets obtained from International Cancer Genome Consortium (ICGC) (https://icgc.org/daco) and The Cancer Genome Atlas (TCGA) (https://cancergenome.nih.gov/), which were processed using *limma* and *vioplot* R software packages. The expression levels of EPDR1 gene in HCC was also explored using the Gene Expression Profiling Interactive Analysis (GEPIA) database (http://gepia.cancer-pku.cn/). Furthermore, we validated the expression of EPDR1 by using three independent Gene Expression Omnibus [23] microarray datasets (GSE76427, GSE64041 and GSE102079) obtained from NCBI‐GEO database (https://www.ncbi.nlm.nih.gov/gds).

### 2.2 Survival analysis using R and Kaplan‐Meier plotter

The prognostic significance of EPDR1 in HCC was explored using the Kaplan‐Meier plotter (http://kmplot.com/analysis/) based on TCGA data and then validated using ICGC database using *survival* package in R software (version 3.5.2). The hazard ratio^30^ with 95% confidence intervals (CI) and log‐rank *P*‐value was also calculated.

### 2.3 Expression level of EPDR1 in human normal tissues

The gene expression and phenotype data corresponding to human normal tissues in GTEx were obtained from UCSC Xena project (http://xena.ucsc.edu/). The downloaded dataset included TOIL RSEM FPKM (n = 7862) and phenotype (n = 9783) data corresponding to different human normal tissues. The Ensembl gene identifiers in the RNA‐seq data were converted to official gene symbols using an in‐house perl script. Further, the expression data of EPDR1 in different organs of males and females were extracted based on GTEx RNA‐seq and phenotype data using perl scripts and visualized using *gganatogram* and *ggpubr* R packages.

### 2.4 Correlation between EPDR1 expression and clinical characteristics of HCC patients

The correlation between EPDR1 expression and different clinicopathological characteristics of HCC patients, such as age, race, gender, survival, histopathological grade, AJCC stage, malignancy, cancer history, and stage was analyzed using R software and then validated using UALCAN (http://ualcan.path.uab.edu). T‐test was used to estimate the significant differences in the gene expression levels between the groups.

### 2.5 Co‐expression analysis using LinkedOmics database

The genes co‐expressed with EPDR1 in HCC were identified using the LinkedOmics database (http://www.linkedomics.org/login.php), a third‐party online tool containing TCGA data. Co‐expressed genes were statistically analyzed and their expression patterns were shown using volcano plots and heatmaps. Heatmaps were generated using the *heatmap* R package. Pearson correlation coefficient was used to evaluate the significant correlation of the co‐expressed genes with EPDR1 expression. FDR < 0.01 was considered for representing significant expression, whereas, *P* < .05 was considered for significantly related genes.

### 2.6 Relationship between EPDR1 expression and immune cell infiltration

The correlation between EPDR1 level and infiltration of immune cells in HCC was explored using Tumor Immune Estimation Resource (TIMER) database (http://cistrome.org/TIMER/). Correlation analysis of EPDR1 expression and infiltration levels of 22 immune cells was further performed based on TCGA HCC data using CIBERSORT (https://cibersort.stanford.edu/). Further, the R package *corrplot* was used to investigate the correlations between the expression pattern of EPDR1 and known immune marker genes in HCC. The EPDR1 expression in infiltrating T cells of liver cancer were analyzed using the single‐cell RNA‐seq datasets (http://hcc.cancer-pku.cn/, Landscape of Infiltrating T Cells in Liver Cancer Revealed by Single‐Cell Sequencing). However, EPDR1 is not detected in immune cell clusters in liver cancer patients. Furthermore, another HCC single‐cell dataset, GSE124395 (Dominic Grün. et.al. A Human Liver Cell Atlas Reveals Heterogeneity and Epithelial Progenitors, nature, 2019) were downloaded to analyzed the expression of EPDR1. Perl was used to obtain EPDR1 expression in various immune cell clusters of HCC patients.

### 2.7 EPDR1 protein levels in HCC

The expression of EPDR1 protein in HCC was explored based on the immunohistochemistry (IHC) data from Human Protein Atlas (HPA) database (https://www.proteinatlas.org/), as well as the mass spectrometric data from Clinical Proteomic Tumor Analysis Consortium (CPTAC) database (https://cptac-data-portal.georgetown.edu/cptacPublic/).

### 2.8 Statistical analysis

Statistical analyses for TCGA data were performed using different packages in R (version 3.6.1). The correlations between clinical characteristics and EPDR1 expression were analyzed using logistic regression. Univariate and multivariate Cox regression analyses were performed to reveal the relationship between EPDR1 and the clinical parameters and the immune cell infiltration with overall survival of HCC using *survival* package in R software. Time‐dependent receiver operating characteristic (ROC) curves, with AUC values were quantified with the survival ROC package. The values with *P* < .05 were considered statistically significant.

## 3 RESULTS

### 3.1 EPDR1 expression is significantly elevated in HCC tissues

**Figure 1:**
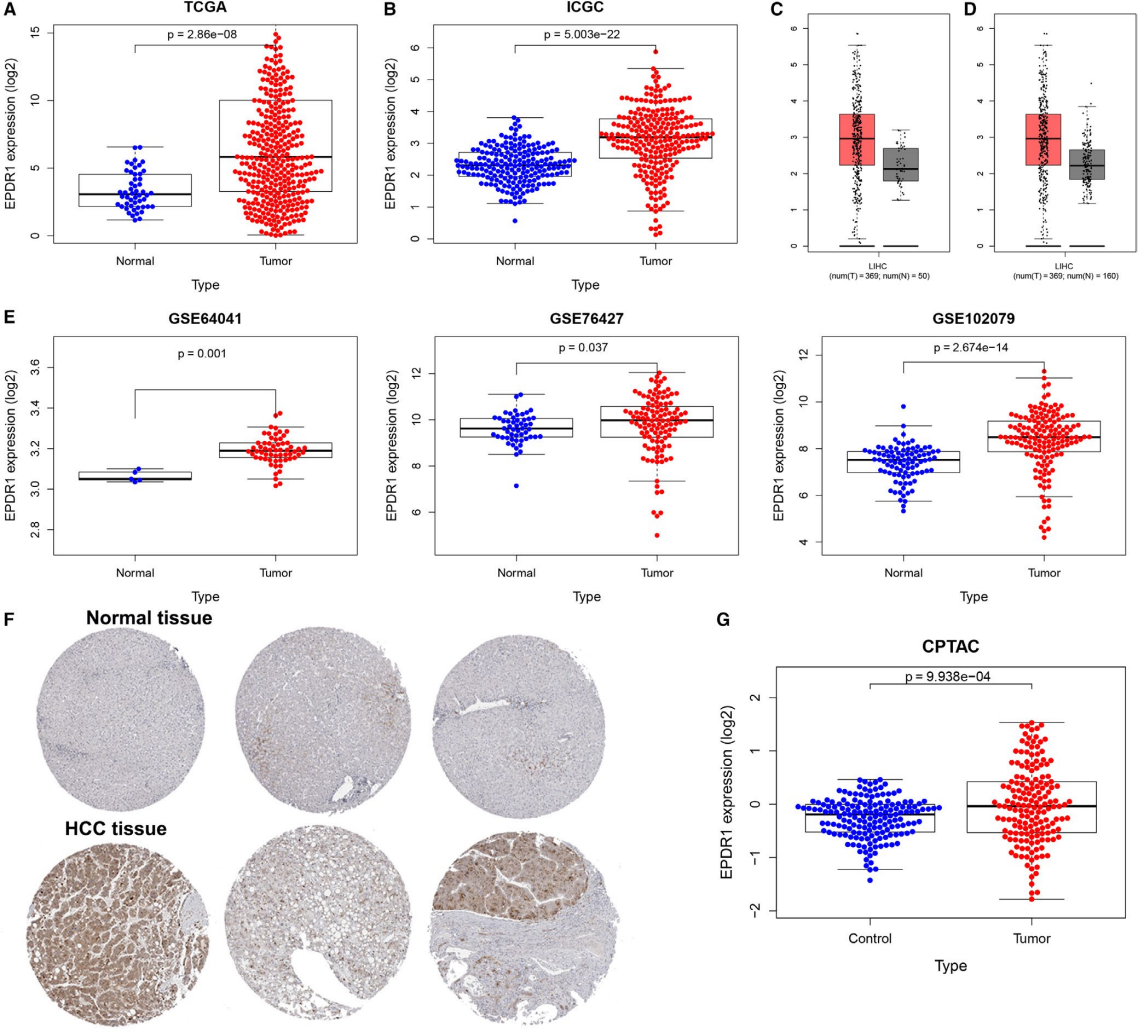
EPDR1 expression in HCC. EPDR1 expression in HCC tissues compared with normal tissues using TCGA database (A) and ICGC database. (B) EPDR1 expression in HCC tissues compared with normal tissues (C) or normal and GTEx data (D) using the GEPIA database. (E) EPDR1 expression in HCC tissues compared with normal tissues using the GEO database. (F) The protein levels of EPDR1 in HCC from HPA database and CPTAC database (G)

First, we compared the expression of EPDR1 in HCC and normal liver tissues using multiple datasets obtained from TCGA, ICGC, GEPIA and GEO databases. Analysis of several HCC cohorts in TCGA and ICGC databases revealed that EPDR1 mRNA expression was significantly higher in HCC tissues than in the adjacent normal tissues (Figure 1A and B. Further, the expression data in the GEPIA database indicated up‐regulation of EPDR1 in the HCC tissues compared to the normal tissues from TCGA (Figure 1C) or GTEx database (Figure 1D). We also compared the expression of EPDR1 in tumor and paracancerous tissues from the same patient (Figure S1). The expression of ERDR1 was significantly higher in tumor *versus* paired normal tissues (*P* < .0001). Additionally, three independent microarray studies obtained from the GEO database validated the significant higher expression of EPDR1 in tumor tissues than in the normal tissue (Figure 1E).

Finally, we verified the protein levels of EPDR1 in HCC tissues using HPA and CPTAC databases. The IHC data in HPA database revealed that the expression of EPDR1 was significantly higher in HCC tissue than the normal tissues (Figure 1F). Further, EPDR1 level was found to be significantly increased in the HCC group compared to that in normal group (*P* = 9.938‐e04) based on CPTAC data, as shown in Figure 1G.

### 3.2 Prognostic significance of EPDR1 expression in HCC

**Figure 2:**
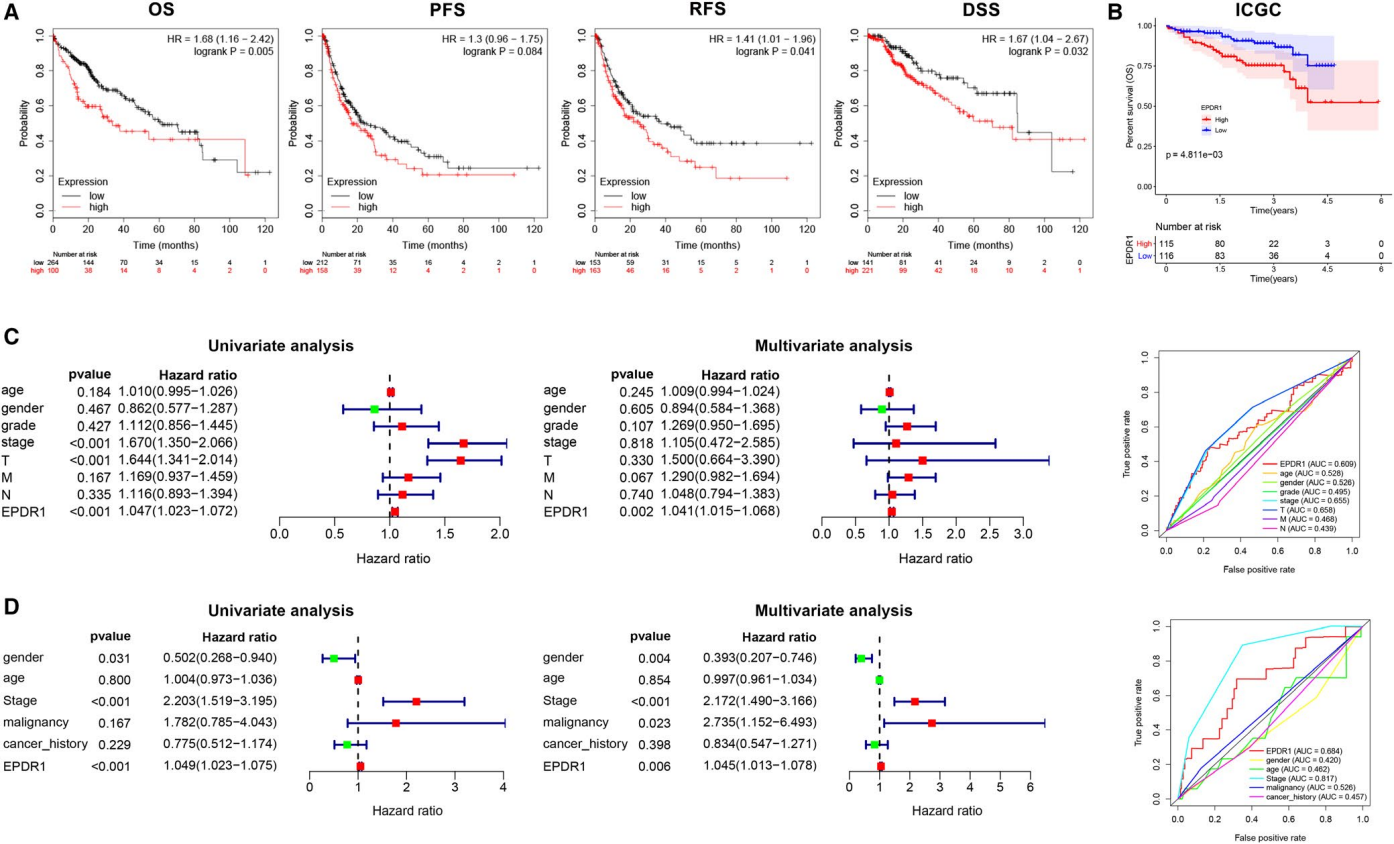
Prognostic significance of EPDR1 expression in human hepatocellular carcinoma (HCC). (A) Correlation between EPDR1 expression and survival of HCC patients (OS, overall survival; FS, progression free survival; RFS, recurrence free survival; DSS, disease specific survival) using Kaplan‐Meier Plotter database. (B) Correlation between EPDR1 expression and overall survival of HCC patients from the ICGC database. Univariate and multivariate Cox regression analysis and ROC curve revealed the relationship between EPDR1 and the clinical factors with overall survival for HCC in the (C) TCGA and (D) ICGC databases

Next, we sought to investigate the prognostic significance of EPDR1 expression in HCC. The association between EPDR1 level and the survival outcomes of HCC patients was assessed using Kaplan‐Meier survival curves (Figure 2). The results showed clustering of patients into two groups according to the median value of EPDR1 expression level in each cohort. The group with high EPDR1 expression had significantly shorter overall survival (OS), progression‐free survival (PFS), recurrence free survival (RFS) and disease specific survival (DSS) compared to the group with low expression of EPDR1 based on TCGA data using GEPIA database (Figure 2A). Figure 2B indicates significant correlation (*P* < .001) between high EPDR1 mRNA expression and worse OS for HCC patients based on the data obtained from ICGC database. As shown in Figure 2C, univariate analysis using Cox regression revealed that EPDR1 expression and T stage are significantly associated with overall survival (*P* < .05). Multivariate analysis and ROC curve revealed that up‐regulated expression of EPDR1 is an independent prognostic factor of poor prognosis based on TCGA data (*P* < .005). The prognostic significance of EPDR1 verified using the data obtained from ICGC database is shown in Figure 2D.

### 3.3 Correlation between EPDR1 expression and clinical characteristics of HCC patients

**Figure 3:**
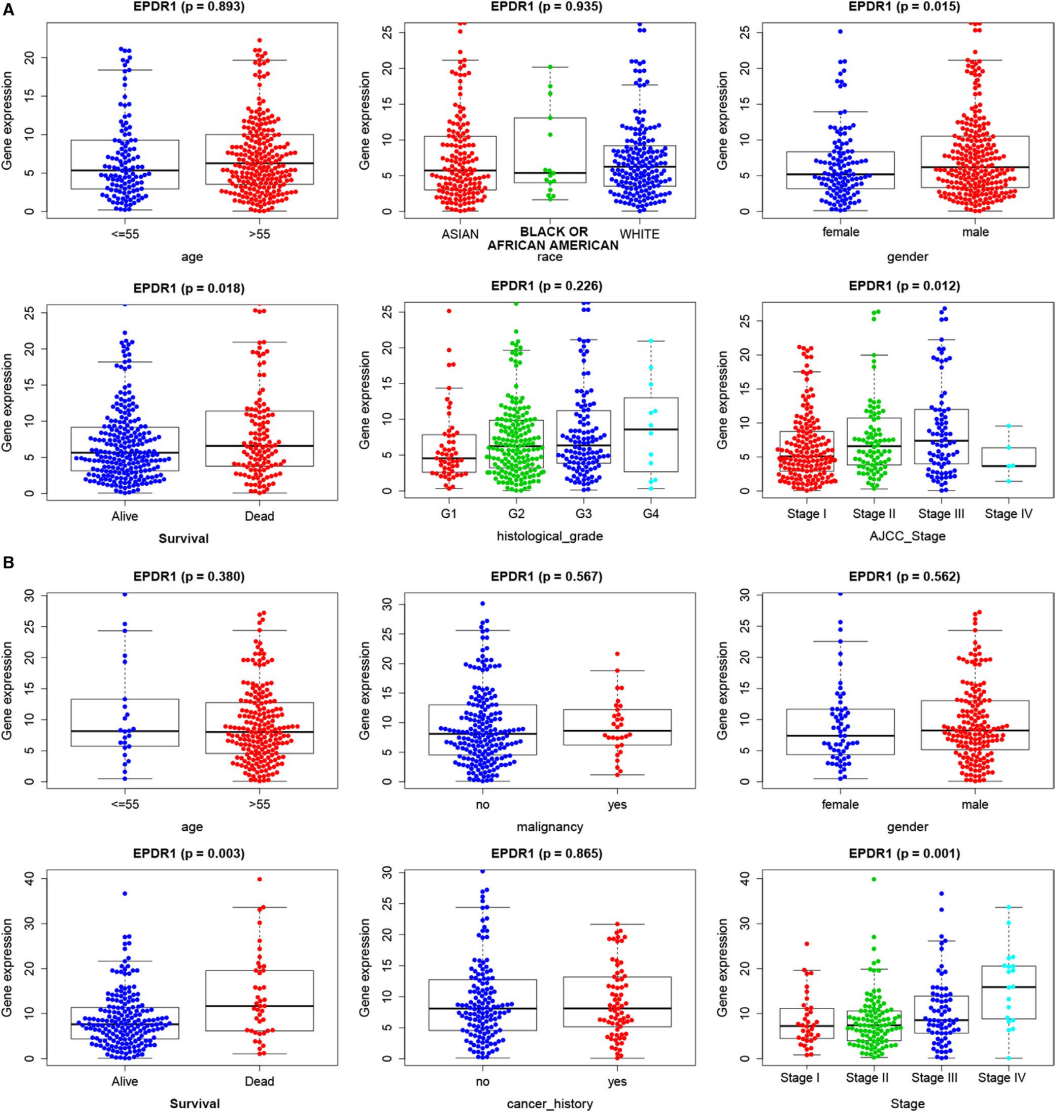
EPDR1 is overexpressed and positively associated with higher tumor stage in human hepatocellular carcinoma (HCC) samples. The expression levels of EPDR1 mRNA are shown across various clinical and pathological stages using TCGA (A) and ICGC (B) databases

The correlation between EPDR1 expression and various clinicopathological parameters in HCC patients based on TCGA and ICGC data are shown in Figure 3A,B, respectively. We observed significantly higher levels of EPDR1 in male patients (*P* = .015), and in patients with advanced stage of the disease (*P* = .018) and bad prognosis (*P* = .012). Furthermore, the expression of EPDR1 was shown to be significantly increased gradually from stage I to stage IV (*P* = .001), and was found to be significantly higher in patients with follow‐up death (*P* = .003). We further compared the expression of EPDR1 considering age, gender, race, weight, grade, stage, histology subtypes, metastasis status and stage of HCC patients (Figure S2). Of note, EPDR1 expression was higher in stage I–IV patients compared to healthy individuals. We also assessed and compared the overall survival of the EPDR1‐high‐expression and EPDR1‐low‐expression groups, per stage, grade, and T of HCC patients (Figure S3). In stage III, grade III and T3 groups, the overall survival was significantly worse in EPDR1‐high‐expression compared to EPDR1‐low‐expression patients (*P* < .05). Thus, these results indicate that EPDR1 is overexpressed and positively associated with advanced tumor stage in HCC.

### 3.4 Expression of EPDR1 in human normal tissues

**Figure 4:**
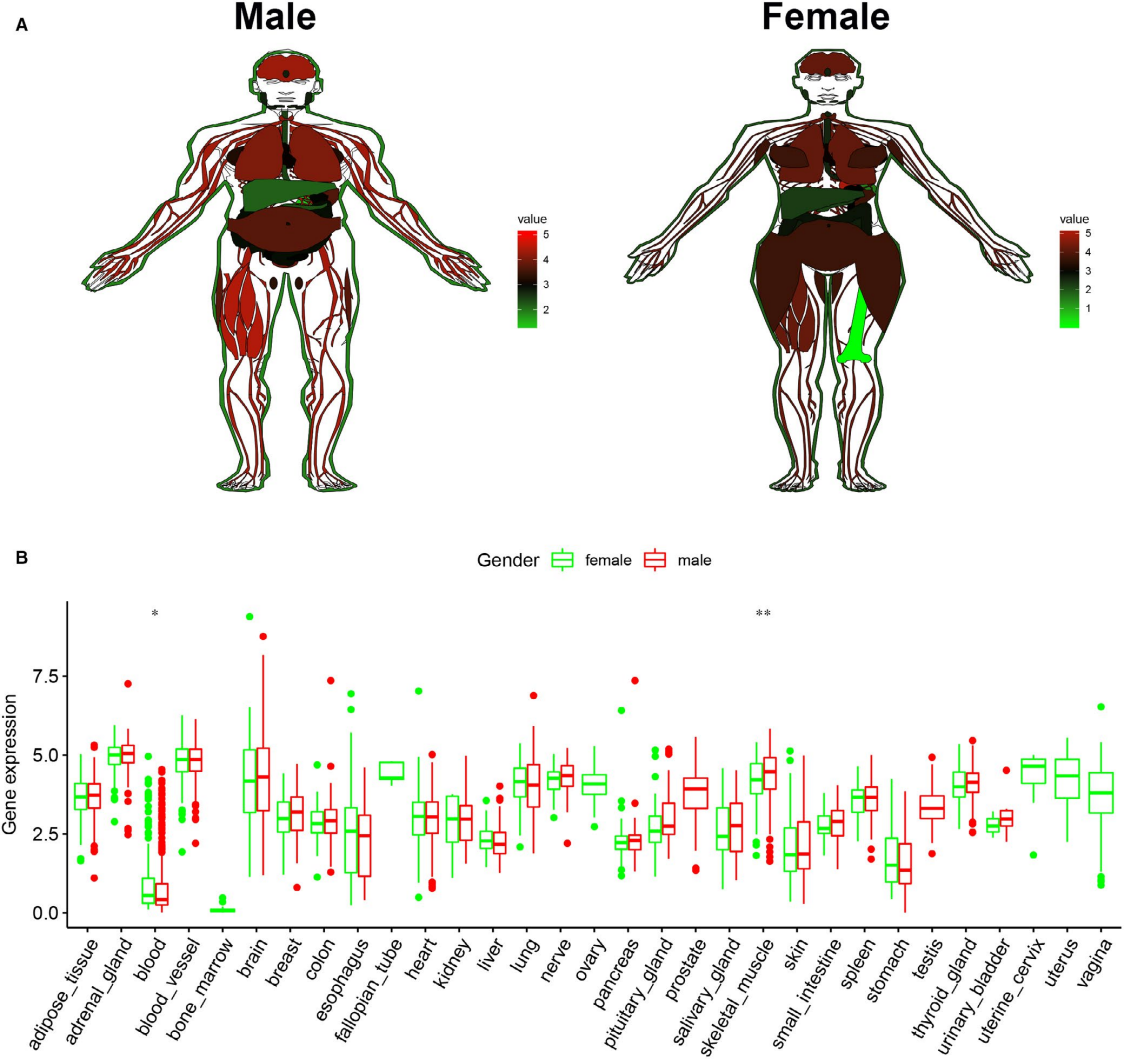
Expression level of EPDR1 in normal human tissues based on the GTEx data. (A) Expression of EPDR1 in male and female tissues. Red represents high expression, black represents median expression, and green represents low expression. (B) Comparative analysis of EPDR1 expression level between male and female tissues

EPDR1 expression in normal human tissues was determined using the data obtained from the GTEx database. Red represents high expression, black represents median expression, and green represents low expression (Figure 4A). EPDR1 was found to be highly expressed in adrenal gland, blood vessel, lung, and nerve tissues, among others. However, the blood, and stomach, pancreas and skin tissues showed EPDR1 expression levels similar to those of liver tissues, as represented in Figure 4A and B. Further, no significant gender‐based difference was observed in the expression of EPDR1 in most tissues or organs, except in case of blood and skeletal muscle (*P* < .05 and *P* < .01, respectively), Figure 4B.

### 3.5 EPDR1 co‐expressed genes in HCC

**Figure 5:**
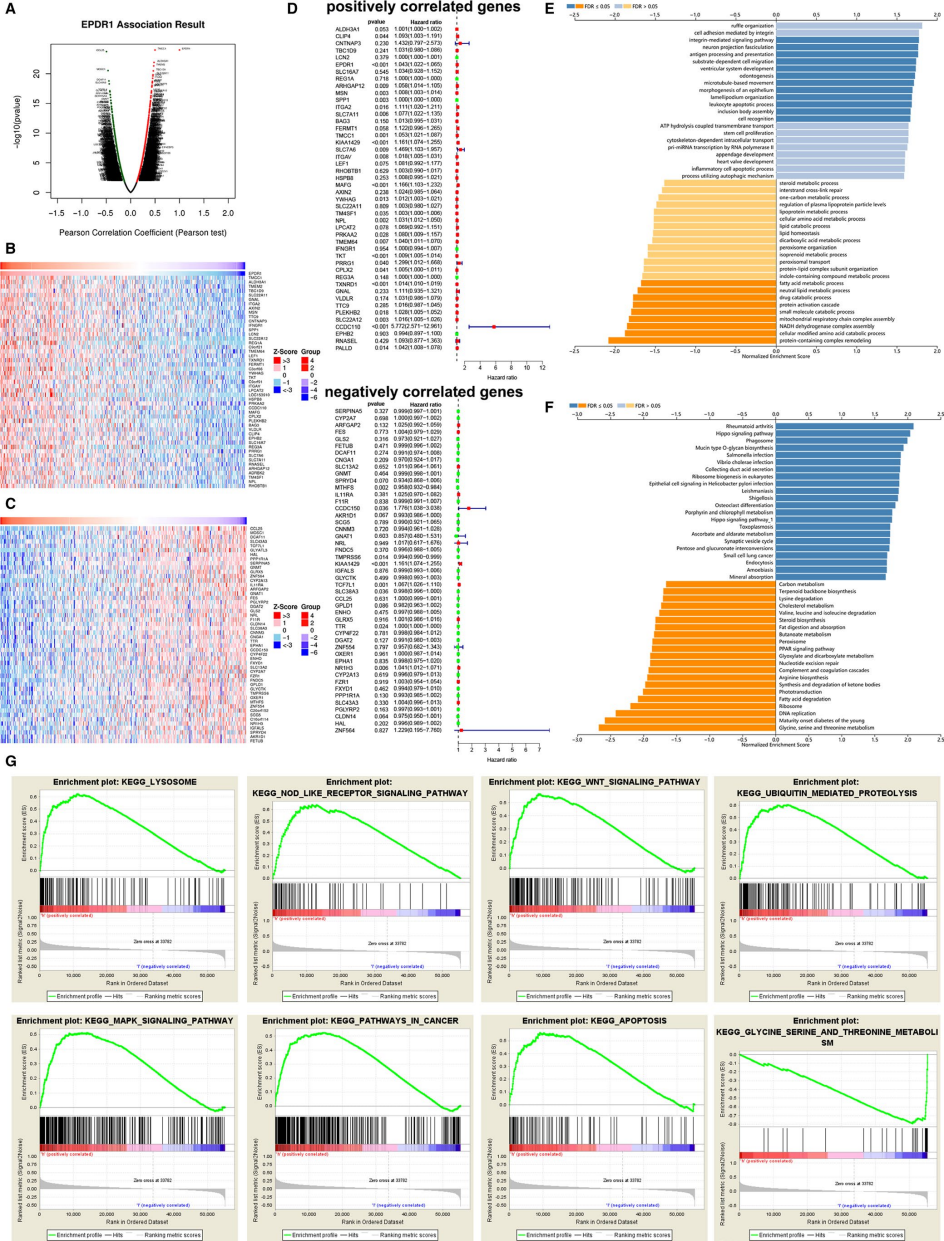
EPDR1 co‐expression genes in HCC (LinkedOmics). (A) The global EPDR1 highly correlated genes identified by Pearson test in HCC cohort. The top 50 genes positively (B) and negatively (C) relative to EPDR1 in HCC were showed by heat maps and survival map (D). GO annotations (E) and KEGG pathways (F) of EPDR1 in HCC cohort. (G) Gene set enrichment analysis plots

To gain insight into the biological importance of EPDR1 in HCC, the function module of LinkedOmics was used to examine the EPDR1 co‐expression in liver HCC (LIHC) cohort. Figure 5A shows genes highly co‐expressed with EPDR1 based on Pearson correlation; genes positively and negatively correlated with EPDR1 are marked in dark red and dark green dots, respectively (FDR < 0.01). Top 50 genes showing significant positive and negative correlation with EPDR1 are shown in heatmaps (Figure 5B and C). The prognostic role of the top 50 genes positively and negatively correlated with EPDR1 in LIHC cohort are shown in Figure 5D. Further, 24 of the top 50 positively correlated genes were shown to be high‐risk genes; whereas 8 of the top 50 negatively correlated genes were low‐risk genes (Figure 5D). Gene Ontology (GO) enrichment analysis showed that EPDR1 co‐expressed genes were significantly associated with the activation of integrin‐mediated signaling pathway, antigen processing, and presentation and leukocyte apoptotic process, while the processes, such as fatty acid metabolism, protein activation cascade were inhibited (Figure 5E). KEGG pathway analysis showed enrichment of pathways such as, hippo signaling, pathways related to various infections, small cell lung cancer, nucleotide excision repair, and DNA replication (Figure 5F). These results suggest a widespread impact of EPDR1 expression on the global transcriptome of HCC tissues.

To further analyze the biological functions of EPDR1 in HCC, GSEA was performed on datasets with high and low expression of EPDR1. These results revealed significant differences (FDR *q*‐value < 0.01) in the enrichment of MSigDB Collection (c2.cp.biocarta and h.all. v6.1. symbols). The most markedly enriched signaling pathways screened according to their NES are shown in Figure 5G. As have been shown, pathways related to lysosome, NOD like receptor signaling, WNT signaling, ubiquitin mediated proteolysis, MAPK signaling, cancer and apoptosis were significantly represented by EPDR1 high expression phenotype, whereas, pathways associated with glycine, serine and threonine metabolism were represented by EPDR1 low expression phenotype. One study on colorectal carcinoma demonstrated that DNA methylation might play a critical role in EPDR1 expression‐regulation.^29^ However, no regulators of EPDR1 were identified so far. Since IDH1/H2 and TP53 mutations are found in a large percentage of HCC patients,^31^ we further analyzed the correlation between IDH1, IDH2, and p53 mutations and the expression of EPDR1 in HCC using TCGA database (Figure S4). No significant correlation was observed between EPDR1 expression and IDH1, IDH2, or p53 mutations.

### 3.6 EPDR1 expression and tumor‐infiltrating immune cells (TIICs)

**Figure 6:**
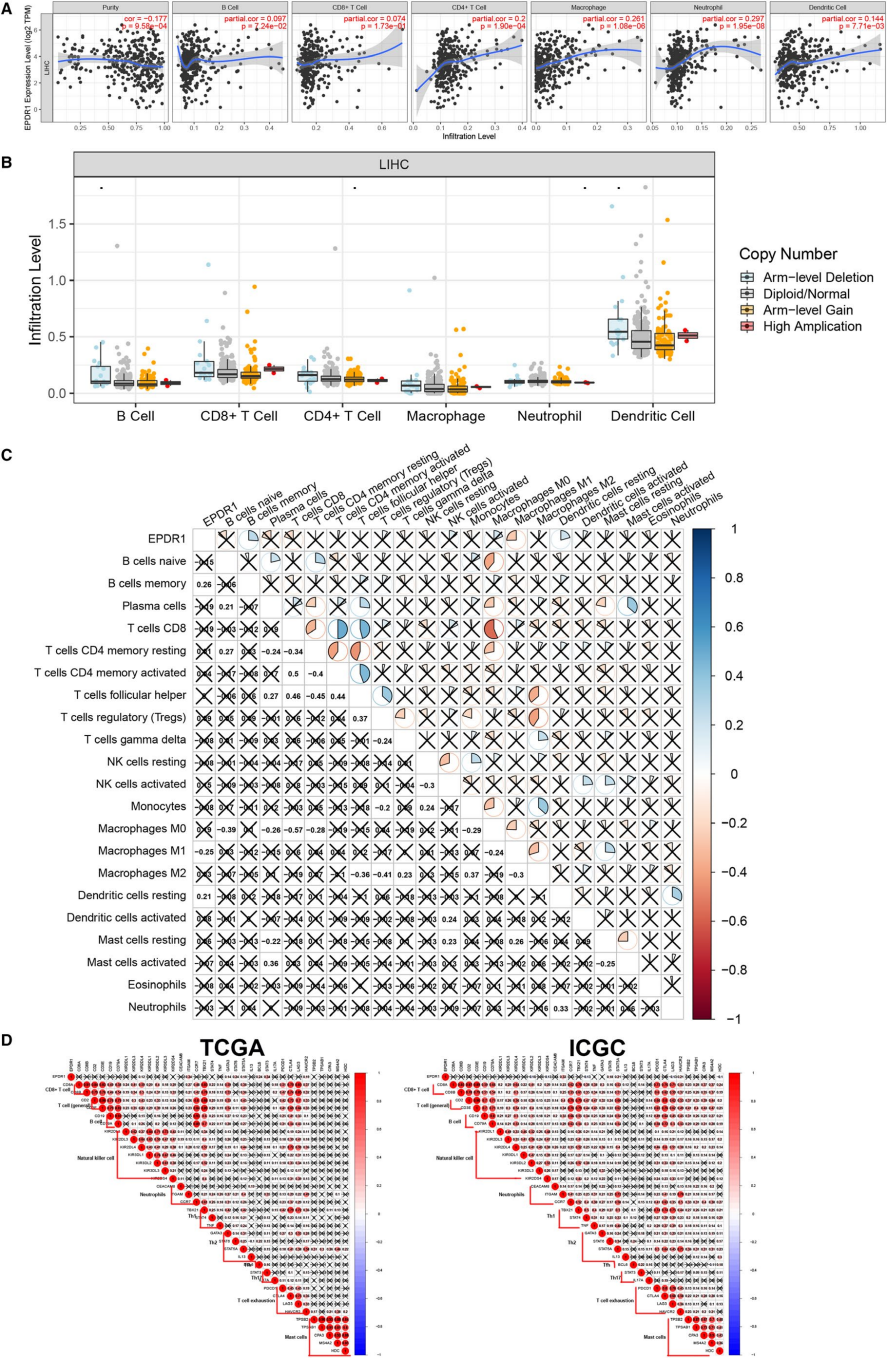
The relationship between EPDR1 and immune cell infiltrations. A, Correlation analysis of EPDR1 expression and infiltration levels of immune cells in HCC tissues using the TIMER database. B, EPDR1 CNV affects the infiltrating levels of B cells, CD4 + T cells, neutrophils, and dendritic cells in HCC. C, Correlation analysis of EPDR1 expression and infiltration levels of 22 immune cells in HCC tissues from TCGA using CIBERSORT. D, The relationship between EPDR1 and immune related genes in TCGA and ICGC

The survival of patients in several cancers is determined by the number and activity of tumor‐infiltrating lymphocytes.^32^ Therefore, we explored the relationship between EPDR1 expression and immune cell infiltration in HCC using the TIMER database. Figure 6A shows that EPDR1 expression significantly correlates with purity, and infiltration of B cells, CD8 + T cells, CD4 + T cells, macrophages, neutrophils and dendritic cells in HCC tissues. Moreover, copy number variation of EPDR1 was shown to affect the infiltration of B cells, CD4 + T cells, neutrophils, and dendritic cells in HCC (Figure 6B). Further, the association between EPDR1 expression and level of infiltration of 22 immune cells in TCGA HCC datasets using CIBERSORT indicated clear infiltration of the immune cell subpopulations. The correlation heatmap (Figure 6C) revealed the proportions of different TIIC subpopulations that were weakly to moderately correlated with the expression of EPDR1.

To better broaden the understanding of EPDR1 crosstalk with immune related genes, we analyzed the association between EPDR1 expression and various immune signatures from TCGA and ICGC datasets. The signatures include immune marker genes of common tumor‐infiltrating lymphocytes (TILs), T cell exhaustion, immune inhibitory or stimulatory genes (including immune checkpoint gene sets), cytokine‐related genes, major histocompatibility complex genes, and mast cells (Figure 6D). The results showed that expression of KIR2DL4 (natural killer cell), ITGAM (neutrophil), GATA3 (TH2), STAT6 (TH2), STAT5A (TH2), BCL6 (Tfh), STAT3 (Th17) and HAVCR2 (T cell exhaustion) were significantly correlated with EPDR1 expression levels based on TCGA database (*P* < .05), while KIR2DL2 (natural killer cell), KIRDS4 (natural killer cell), ITGAM (neutrophil), STAT4 (Th1), STAT5A (Th2), STAT6 (Th2), LAG3 (T cell exhaustion), HAVCR2 (T cell exhaustion) and HDC (mast cells) were significantly correlated with EPDR1 expression levels based on ICGC database (*P* < .05).

## 4 DISCUSSION

HCC is the most common type of liver cancer. Most HCC patients are diagnosed at an advanced stage, which lacks effective treatment.^33^, ^34^ Therefore, early screening and diagnosis of HCC patients is beneficial for the treatment and prognosis of patients. However, currently there are no effective biomarkers for the early diagnosis of HCC, and the molecular mechanisms underlying HCC metastasis remains unclear.^35^, ^36^ EPDR1, a member of piscine brain glycoproteins, plays a role in cell adhesion. To gain detailed insights into the potential functions of EPDR1 and its regulatory network in HCC, we performed bioinformatics analysis using publicly available data, which we hope will benefit future research related to HCC.

In the current study, we explored the expression of EPDR1 in HCC patient data obtained from various database to assess its prognostic value. High expression of EPDR1 was significantly correlated with worse RFS, PFS, OS, and DSS of HCC patients. Further, the relationship between EPDR1 expression and various clinicopathological characteristics of HCC patients highlights the role of elevated EPDR1 as an independent prognostic factor for poor OS. The HCC patients with high EPDR1 expression are more likely to present advanced grade, stage, and poor prognosis than those with low EPDR1 expression. Therefore, our findings suggest that EPDR1 overexpression occurs in most HCC cases and deserves further clinical validation as a potential diagnostic and prognostic marker.

To probe the signaling pathways associated with EPDR1 expression, we constructed the EPDR1 co‐expression network and performed functional analysis of the co‐expressed genes. GO and KEGG pathway analysis revealed that the co‐expressed genes were mainly related to processes and pathways associated with different signaling, DNA replication and metabolism. To further analyze the biological role of EPDR1 in HCC, GSEA was performed, which revealed that EPDR1 overexpression was implicated in multiple signaling pathways, such as WNT, MAPK, NOD like receptor, and cancer, and apoptosis. These signaling pathways and processes have been reported to be associated with HCC carcinogenesis.^37^ However, in vivo studies are needed to further validate the association of these processes and pathways in regulating EPDR1 functions in HCC.

The liver is the largest immune organ of the body and harbors various immune cells. HCC is a typical inflammatory related tumor, and immune tolerance and escape plays a key role in the carcinogenesis process.^38^ Previous studies have mainly focused on tumor proliferation and invasion, whereas, recently the concept of tumor immune microenvironment has emerged as one of the important factors. In tumor microenvironment (TME), cancer cells, non‐cancerous cells, and extracellular matrix form a dynamic system that interacts with each other. A number of growth factors, cytokines and chemokines are produced in the TME that facilitate immune tolerance, and promote progression of cancer.^39^ Therefore, we explored the potential role EPDR1 in immune regulation in HCC using various databases. Our study indicated that EPDR1 expression significantly correlates with the infiltration levels of various immune cells in the TME of HCC, suggesting a role of EPDR1 in modulating cancer immunity. Further, our results indicated a possible mechanism, where EPDR1 regulates the functions of different immune cells in HCC. The analysis revealed significant correlation of several immune signatures with EPDR1 level. Most cancers evade immune supervision by impairing the functions of immune cells, such as tumor associated neutrophils, regulatory T cells, marrow‐derived suppressor cells, tumor‐associated macrophages and/or by releasing inhibitory cytokines, such as GM‐CSF, VEGF, IL‐1β and chemokines.^16^, ^39^, ^40^, ^41^ These immune cells and cytokines are vital players of TME and are associated with immune regulation in HCC. Hence, exploring these factors might provide novel targets for immunotherapy of HCC. We also analyzed EPDR1 expression in different immune cell clusters using two single‐cell RNA‐seq datasets.^42^, ^43^ Since the results showed that EPDR1 expression was very low in various immune cell clusters (Figure S5), we speculate that HCC cells‐derived EPDR1 might modulate immune cell infiltration into the tumor microenvironment in a paracrine manner. To summarize, our results indicate that EPDR1 plays a crucial role in the modulation and recruitment of immune cells as well as in affecting the expression of immune signatures in HCC. However, further research, including in vivo and in vitro validation, as well as clinical trials with optimum numbers of samples, are needed to evaluate the correlation between EPDR1 and immune regulation more accurately in HCC.

In conclusion, our study identifies EPDR1 as a novel biomarker with prognostic significance in HCC patients. Our results indicate an association between EPDR1 expression and immune cell infiltration and unveil the potential molecular mechanism underlying the carcinogenesis in HCC. Thus, we conclude that EPDR1 can be used as an effective tool or target for the diagnosis or treatment of HCC, respectively, in the future.

## CONFLICT OF INTEREST

The authors declare that there are no conflicts of interest.

## AUTHOR CONTRIBUTION

**Ruochan Chen:** Conceptualization (equal); Data curation (equal); Formal analysis (equal); Funding acquisition (equal); Investigation (equal); Methodology (equal); Project administration (equal); Resources (equal); Software (equal); Supervision (equal); Validation (equal); Visualization (equal); Writing‐original draft (lead); Writing‐review & editing (equal). **Yiya Zhang:** Conceptualization (equal); Data curation (equal); Formal analysis (lead); Funding acquisition (equal); Investigation (equal); Methodology (equal); Project administration (equal); Software (lead); Supervision (equal); Validation (equal); Visualization (equal); Writing‐original draft (supporting); Writing‐review & editing (equal).

## Supporting information


**Figure S1–S5**

## Data Availability

The data that support the findings of this study are available in multiple databases and repositories. These data were derived from the following resources available in the public domain: International Cancer Genome Consortium (ICGC) (https://icgc.org/daco); The Cancer Genome Atlas (TCGA) (https://cancergenome.nih.gov/); Gene Expression Profiling Interactive Analysis (GEPIA) database (http://gepia.cancer-pku.cn/); Gene Expression Omnibus [23] database (https://www.ncbi.nlm.nih.gov/gds); Kaplan‐Meier plotter (http://kmplot.com/analysis/); UCSC Xena project (http://xena.ucsc.edu/); UALCAN (http://ualcan.path.uab.edu); LinkedOmics database (http://www.linkedomics.org/login.php); Tumor Immune Estimation Resource (TIMER) database (http://cistrome.org/TIMER/); CIBERSORT (https://cibersort.stanford.edu/); Human Protein Atlas (HPA) database (https://www.proteinatlas.org/); Clinical Proteomic Tumor Analysis Consortium (CPTAC) database (https://cptac-data-portal.georgetown.edu/cptacPublic/).
