# Supplementary material for: EPDR1 correlates with immune cell infiltration in hepatocellular carcinoma and can be used as a prognostic biomarker
Source: J Cell Mol Med. 2020 Sep 15;24(20):12107–18. doi: 10.1111/jcmm.15852 (PMC7579695; doi:10.1111/jcmm.15852)
Supplement: Supplementary file 1 — Figure S1–S5 [file JCMM-24-12107-s001.docx]

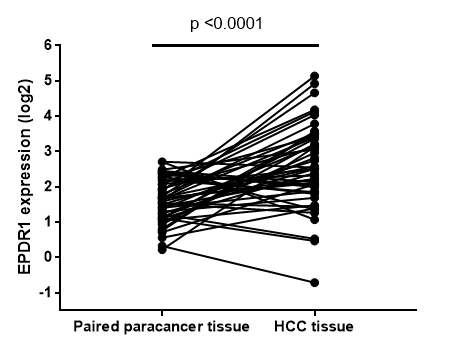


Supplementary Figure 1 EPDR1 expression in HCC


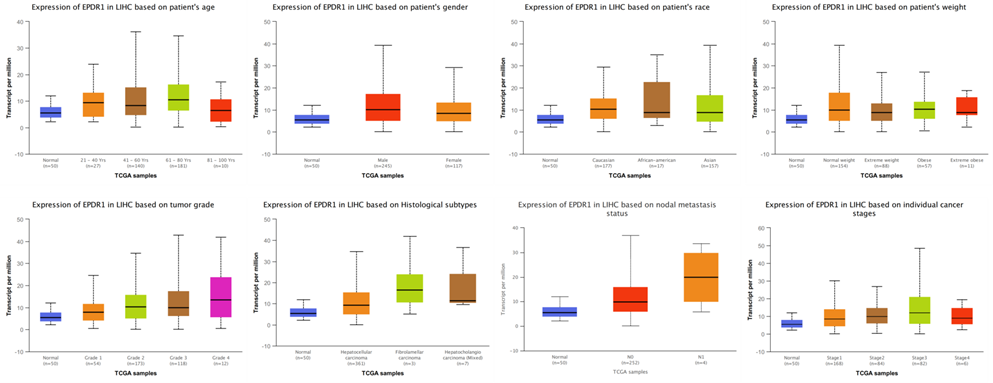


Supplementary Figure 2 EPDR1 expression in different age, gender, race, weight, grade,stage, histology subtypes, metastasis status and stage of HCC patients

The statistical significance between groups is shown in table below

Table Statistical significance between groups

1. Age

| Comparison | Statistical significance |
| --- | --- |
| Normal-vs-Age(21-40Yrs) | 1.264020E-02 |
| Normal-vs-Age(41-60Yrs) | 5.87209947333633E-10 |
| Normal-vs-Age(61-80Yrs) | 1.62447832963153E-12 |
| Normal-vs-Age(81-100Yrs) | 1.367850E-01 |
| Age(21-40Yrs)-vs-Age(41-60Yrs) | 7.341800E-01 |
| Age(21-40Yrs)-vs-Age(61-80Yrs) | 7.964000E-01 |
| Age(21-40Yrs)-vs-Age(81-100Yrs) | 7.840000E-01 |
| Age(41-60Yrs)-vs-Age(61-80Yrs) | 9.215600E-01 |
| Age(41-60Yrs)-vs-Age(81-100Yrs) | 8.864600E-01 |
| Age(61-80Yrs)-vs-Age(81-100Yrs) | 8.319600E-01 |

1. Gender

| Comparison | Statistical significance |
| --- | --- |
| Normal-vs-Male | 1.62436730732907E-12 |
| Normal-vs-Female | 7.50399997695439E-09 |
| Male-vs-Female | 4.958100E-02 |

1. Race

| Comparison | Statistical significance |
| --- | --- |
| Normal-vs-Caucasian | 2.66453525910038E-15 |
| Normal-vs-AfricanAmerican | 1.266410E-02 |
| Normal-vs-Asian | 2.63570276715086E-11 |
| Caucasian-vs-AfricanAmerican | 4.608600E-01 |
| Caucasian-vs-Asian | 4.447200E-01 |
| AfricanAmerican-vs-Asian | 7.886400E-01 |

1. Weight

| Comparison | Statistical significance |
| --- | --- |
| Normal-vs-Normal_Weight | 3.23652216138726E-12 |
| Normal-vs-Extreme_Weight | 2.6582000001163E-07 |
| Normal-vs-Obese | 1.55376999999568E-05 |
| Normal-vs-Extreme_Obese | 3.031600E-02 |
| Normal_Weight-vs-Extreme_Weight | 2.097400E-01 |
| Normal_Weight-vs-Obese | 3.742200E-01 |
| Normal_Weight-vs-Extreme_Obese | 6.077800E-01 |
| Extreme_Weight-vs-Obese | 8.193200E-01 |
| Extreme_Weight-vs-Extreme_Obese | 9.725200E-01 |
| Obese-vs-Extreme_Obese | 8.742600E-01 |

1. Grade

| Comparison | Statistical significance |
| --- | --- |
| Normal-vs-Grade 1 | 3.647900E-04 |
| Normal-vs-Grade 2 | 9.81659198373563E-13 |
| Normal-vs-Grade 3 | 1.1990963777464E-11 |
| Normal-vs-Grade 4 | 2.592400E-02 |
| Grade 1-vs-Grade 2 | 1.352010E-01 |
| Grade 1-vs-Grade 3 | 1.852280E-02 |
| Grade 1-vs-Grade 4 | 2.544800E-01 |
| Grade 2-vs-Grade 3 | 2.265000E-01 |
| Grade 2-vs-Grade 4 | 7.370400E-01 |

1. Histological subtype

| Comparison | Statistical significance |
| --- | --- |
| Normal-vs-Hepatocellular carcinoma | 1.62436730732907E-12 |
| Normal-vs-Fibrolamellar carcinoma | 3.336000E-02 |
| Normal-vs-Hepatocholangio carcinoma (Mixed) | 2.676800E-01 |
| Hepatocellular carcinoma-vs-Fibrolamellar carcinoma | 3.771800E-01 |
| Hepatocellular carcinoma-vs-Hepatocholangio carcinoma (Mixed) | 5.384200E-01 |
| Fibrolamellar carcinoma-vs-Hepatocholangio carcinoma (Mixed) | 9.765000E-01 |

1. Metastasis status

| Comparison | Statistical significance |
| --- | --- |
| Normal-vs-N0 | 1.62447832963153E-12 |
| Normal-vs-N1 | 1.283750E-01 |
| N0-vs-N1 | 5.345800E-01 |

1. Stage

| Comparison | Statistical significance |
| --- | --- |
| Normal-vs-Stage1 | 2.10980122261617E-11 |
| Normal-vs-Stage2 | 1.53779999956249E-07 |
| Normal-vs-Stage3 | 2.53010001838305E-09 |
| Normal-vs-Stage4 | 1.793420E-01 |
| Stage1-vs-Stage2 | 6.717600E-02 |
| Stage1-vs-Stage3 | 1.540810E-03 |
| Stage1-vs-Stage4 | 6.769600E-01 |
| Stage2-vs-Stage3 | 1.855200E-01 |
| Stage2-vs-Stage4 | 4.167600E-01 |
| Stage3-vs-Stage4 | 2.691500E-02 |


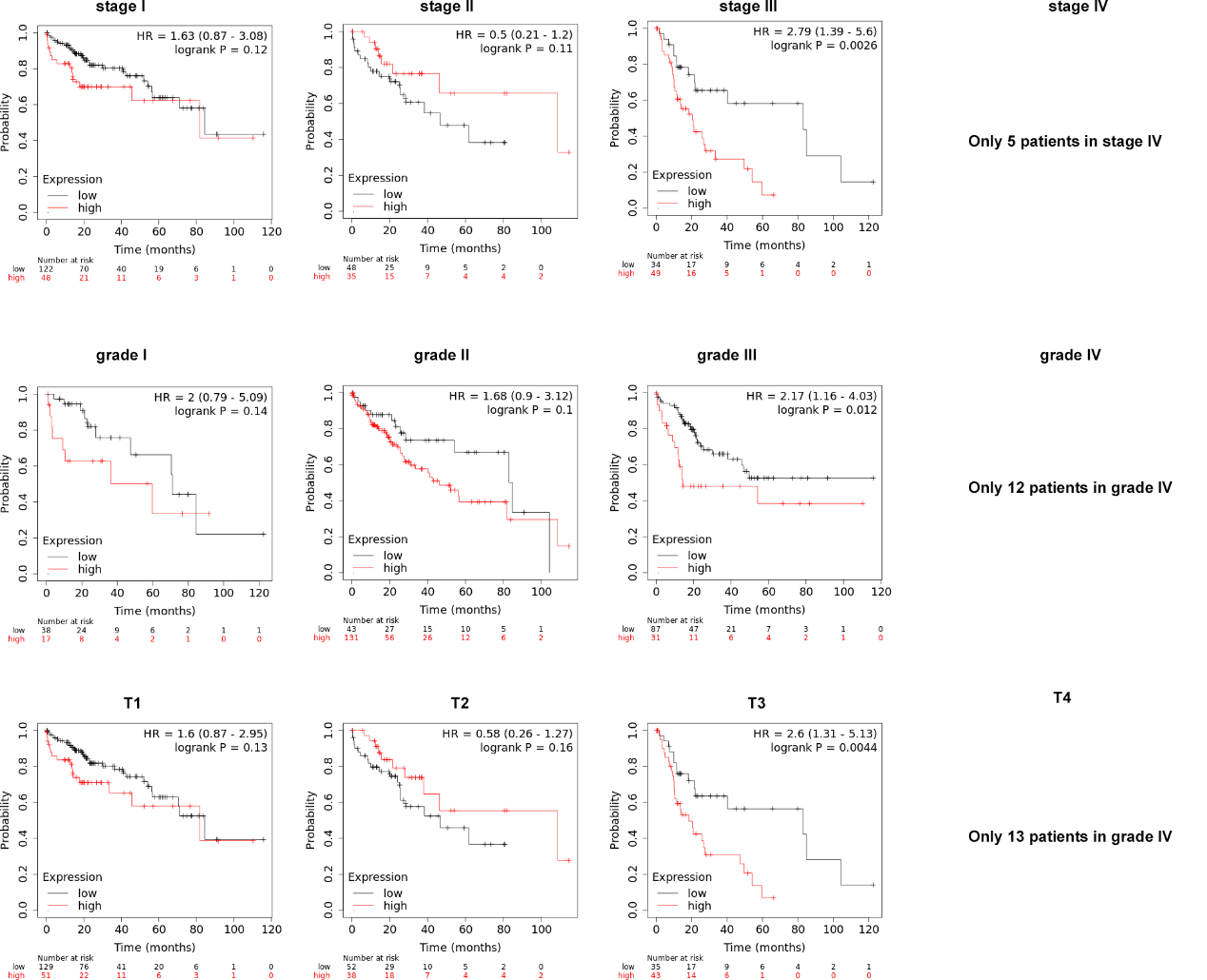


Supplementary Figure 3 the overall survival between EPDR1-high-expression group and EPDR1-low-expression group in different stage, grade and T of HCC patients


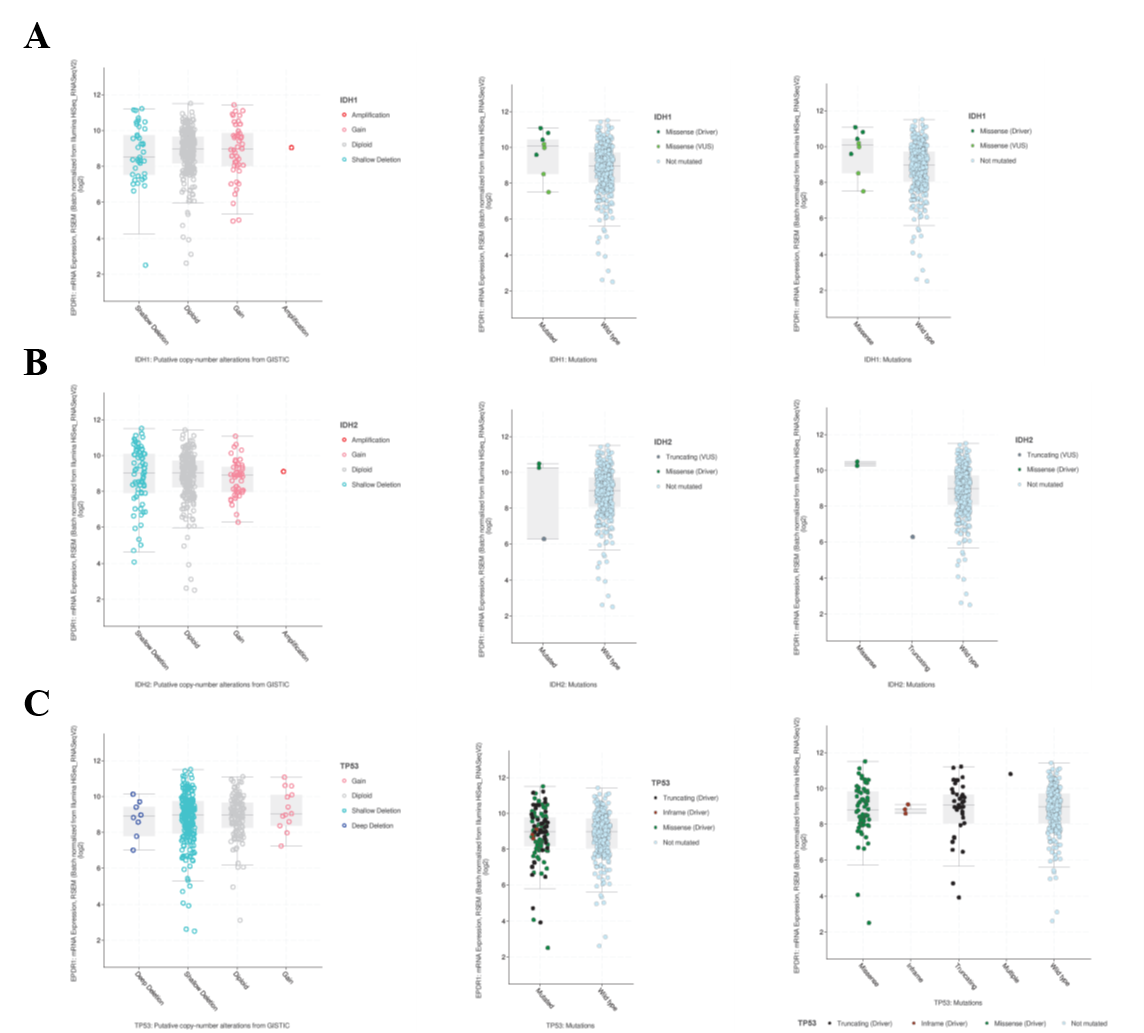


Supplementary Figure 4 The correlation between EPDR1 expression and IDH1 (A), IDH2 (B) and p53 (C) mution in HCC patients.


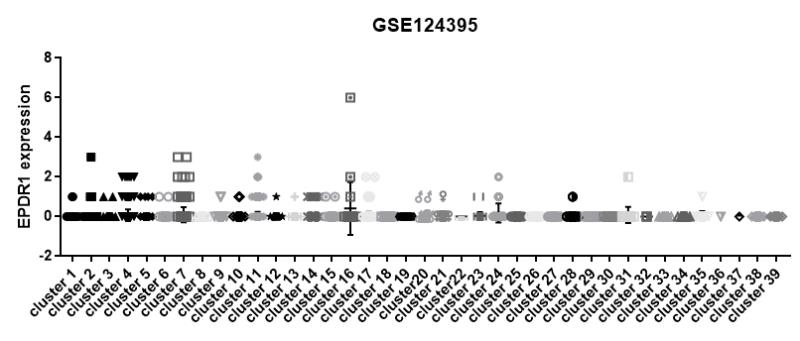


Supplementary Figure 5 EPDR1 expression in various immune cell clusters of HCC patients
